# The Pathology and Treatment of Pulpless Teeth

**Published:** 1894-11

**Authors:** W. H. White

**Affiliations:** Silver City, New Mexico


### THE PATHOLOGY AND TREATMENT OF PULPLESS TEETH.

BY DR. W. H. WHITE, SILVER CITY, NEW MEXICO.

During the past two years I have been using methods of treatment in these cases that are partly original with me, and have had such marked success that I now approach them with far more confidence than I formerly did. But before going into the details of treatment, it is best to get as clear an idea as possible of exactly the conditions we have to treat.

In studying the human body we are too apt to look at it only in the light of an individual,—of an entirety,—just as most people look upon the ocean as something to sail ships on or for fish to live in; but the chemist is more interested in a molecule of water than in an ocean. It is for him to study the attributes of a single molecule, its component parts, its affinities, its likes and dislikes; for when he understands one molecule, he understands every molecule of water in the whole ocean. It is so with the surgeon. He should study the minute component parts of the body, learn their nature, their methods, and their capabilities.

Life begins in a single cell of microscopic size. Our bodies are communities composed of the myriad progeny of this cell. When life first dawned on this planet, they were beings composed of a single cell. These we say were created by divine power, for as yet we have not been able to account for them in any other way. These cells, to a degree, had all the functions that our bodies have, —the sense of sight, touch, nervous and muscular power, the ability to digest and assimilate food; they propagated themselves simply by division, each half becoming a fully-developed being. With the production of these the creative power stopped. All the other phases of life which we see are due to development, to community of interests, to specification, to heredity.

But these small, single cells soon began to have enemies to contend against, and so some varieties gradually discovered that, instead of living alone, they fared better and were better protected living in communities.

Next the laws of heredity and of the physiological division of labor, as expounded by Professor Darwin, began to have their effect on these communities; those cells on the outside of the community began to perfect themselves in methods of warding off the attacks of enemies, while the cells of the interior, relieved of the necessity of self-defence, gradually perfected themselves as muscle-cells, nerve-cells, bone-cells, etc.

All life is simply microbic, the difference being that some live singly, while others live in communities.

The human body is one of these communities; it is in many respects the highest result of this law of the physiological division of labor in the animal kingdom. As a result of these laws our bodies have become aggregations of cells of very varied functions, as an outside covering in the skin and mucous membrane; they have an almost impenetrable armor for the protection of the community; while within they have cells highly developed and highly specialized to perform their allotted functions.

It must be remembered, however, that while these interior cells have thus been developed and specialized, they have at the same time lost at least one very important ability,—namely, that of self-protection. They have gradually lost this from several causes. First, because they are so well protected by the skin and mucous membrane surrounding them; secondly, they are individuals of very sedentary habits. They have slaves to wait on them,—to bring them their food, drink, and fuel. They also have masons and builders to repair any breach in their structure, and soldiers to expel intruders. From these causes they have become very effeminate, and are entirely incapable of defending themselves from their enemies. The red blood-corpuscles are the slaves of this community; they are the "hewers of wood and drawers of water." The white blood-corpuscles are the masons and builders; they are also the soldiers of this human hive.

It is the functions, abilities, and methods of these leucocytes that we should study most carefully, for all that the surgeon can ever do is simply to assist them to do their work. It therefore behooves us to learn thoroughly what these can do; learn the conditions necessary for their successful work and their methods of doing this, so that we may assist and in no way hinder them; learn what their enemies are, and how to remove or destroy these without at the same time injuring them.

Now, in studying these things, suppose a thorn is thrust into the body; what takes place if things are allowed to take their natural course? First, there is irritation to the nerves caused by the thorn. This immediately notifies the sensorium that something is wrong, which, in turn, directs an increased flow of blood to the part. This determination of blood to the part soon fills the vessels there to their utmost capacity, causing congestion and final stagnation of the blood. This is the community's method of fighting its enemies, and it is this ability to concentrate its whole force at one place that makes the community powerful over that of its individual members.

The leucocytes in the normal blood-current move aimlessly along without apparent object or use, as sentinels upon duty. When the part becomes inflamed, they adhere to the walls of the vessel and possess the ability of creeping through the wall into the surrounding tissues, and as they pass through the tissues they leave a canal behind them. This is a new capillary through which they receive sustenance, for they are careful soldiers and do not want their base of supplies cut off. When they reach the tissues, a wonderful change takes place. Under ordinary circumstances the leucocyte would travel along the blood-vessels for an indefinite time as a single individual, but when it reaches the tissues it begins to multiply sometimes with great rapidity. The young leucocytes are packed in and around the thorn, their base of supply is cut off; but this is immaterial, as they increase until the thorn is expelled by pressure of numbers. With this flows a gush of pus. This pus is composed of the bodies of these leucocytes slain in the struggle. This kind of pus is what the surgeons call laudable pus, and well it is so named, for the heroes who defended the pass of Thermopylæ were not more laudable than these.

The vital powers now exude serum which quickly coagulates and forms a scab, then no more pus is formed. The lencocytes now change their function from that of soldiers to that of masons and builders, but instead of using bricks and mortar, they build in their own bodies and the bodies of their children to repair this breach, each one going forward to take its place and leaving a canal (capillary) behind through which it receives its food, until the entire breach is repaired.

But why did the vital powers use such haste to throw out this scab? Why is it that the body is everywhere surrounded by this armor of skin and mucous membrane? The reason is this: in every particle of air, in every drop of water, ever present, ever aggressive, is the *microbe.* There are carnivorous microbes; there are herbivorous microbes; there are lithivorous microbes; there are omnivorous microbes. A microscopic world of which our eyes unaided take no cognizance. Their numbers are myriads, even their varieties are innumerable. A German scientist has lately counted sixteen thousand five hundred microbes to the square inch of material taken from the floor of a first-class railway coach, so that when a person sits in one of these seats, which by the way are supposed to be kept clean, he is surrounded by from one to two hundred million of these minute organisms. From the secretions of the mouth there have been pure cultures made of at least twenty-two distinct varieties of them. No germ of life too pure for their fastidious taste; no loathsome carion too foul to deter them from feasting. These are the ancient and hereditary foes of the human cell. To ward off these was developed this armor of skin and mucous membrane. To keep out these the vital powers hastened to throw out this scab.

But if this scab be disturbed, or is imperfectly formed, then the microbe enters this breach made by the expulsion of the thorn, attacking the young and tender leucocytes, and these cells pass off as pus.

Then the vital forces renew their energy, blood is determined anew to the part, leucocytes are increased, and if the breach is not too deep, or if the microbes have not an opportunity to propagate themselves, then, in spite of thousands of leucocytes being slain, the vital forces usually succeed in repairing the breach. But with a deep pocket that cannot easily be cleaned, then the microbes will multiply wonderfully fast and devour the leucocytes as fast as they appear. The vital powers, after a long and unavailing effort, give up the attempt to repair the breach, and then construct a wall of defence before the healthy cells. This wall of defence has been miscalled a pyogenic membrane, but it is in no sense pus-breeding; it is simply a wall that the vital powers erect to keep the microbes from invading the healthy tissues. Having given up the contest, the vital forces retire behind this wall of defence, the inflammation subsides, the determination of blood to the part ceases.

Here we have a chronic ulcer. In cases where the vital powers are too weak to thoroughly construct this inner wall of defence, the microbes invade the healthy tissues and feed on the highly specialized but effeminate cells of the interior of the body. Then we have a phagedenic ulcer.

At first thought it would seem impossible that the cells of the body could withstand the continued attacks of these microbes, and, in fact, taken singly, the cells of the interior of the body are entirely incapable of contending with them; but it is the great power of community, of interests, the ability of the whole community, to concentrate its fighting force at one place. It is this *vis a tergo*, this so-called vital force, that usually enables the community to come out victorious. At any rate, we know that the human cells have fought these battles during the ages of the past, and have not only come out victorious, but have perfected themselves until they now constitute the most powerful organism that the world has yet produced. In the words which Shakespeare puts into the mouth of Julius Cæsar, "Danger knows full well that Cæsar is more dangerous than he. We were two lions littered in one day, and I the elder and more terrible."

Now, a tooth is but a specialized part of the mucous membrane, and is, therefore, part of this armor which covers and protects the effeminate cells of the interior. When a tooth is decayed and the pulp is destroyed, an opening is made through this armor, allowing the microbes to reach and attack the interior cells. This is the usual natural cause of alveolar abscess. As to treatment, it is in a few words,—destroy the microbes and protect the human cells.

We have many germicides and antiseptics, but the great point to be borne in mind in using these agents is that we have two sets of germ-life here side by side; the one it is necessary to destroy, the other it is just as necessary *not* to destroy, but to keep alive and healthy. As an illustration of what we should accomplish, it is known, for instance, that Persian insect powder is entirely harmless to lung-breathing animals, but is deadly poison to insects.

The germicide that should be used is one that would kill the microbe, but be entirely innocuous to the human cell. Creosote and carbolic acid are good germicides, but they are also cauterants; they kill the microbes but also kill the human cell. Bichloride of mercury is a good germicide, but it is poisonous to the human cell; it is also a coagulant of albumen, and, meeting the serum of the blood in a fine pulp-canal, coagulates the albumen, makes a dam, thus defeating its purpose of reaching the microbe in the apical space. Oil of cloves is an irritant. Iodoform is not sufficiently penetrating.

The properties of the agent we use should be (1) germicidal, (2) non-coagulant, (3) penetrating, (4) non-cauterant, non-irritant. The oil of cinnamon so nearly meets all these requirements, and is so effective in practice, that I have discarded all other remedies in the treatment of these cases. It also has the virtue of producing a pleasant odor in the office instead of a disagreeable one.

Having curèd the ulcer, the next step is to so fill the root-canal as to effectually keep out the microbes. At first thought anything that would perfectly seal the canal would answer the purpose, but practically it is found that in some teeth with crooked roots, and, in fact, in most teeth, it is practically impossible to clean and fill perfectly entirely to the apical foramen, and when we do our best there is more or less of the canal near the apex that is not perfectly filled. Our root-filling may protect this part of the canal from germs from without, but it may not protect it from germs from within. I believe there are some varieties of germs in the blood which have the nature of a ferment. They do not attack living cells, therefore they are tolerated within the system; but coming to the apical foramen, they find dead matter and begin to devour it. They exhale gases which soon become a mechanical irritant. These germs do not kill leucocytes and thus form pus, but the pus formed in these cases is of the laudable variety; it is leucocytes killed in the attempt to expel this gas. This is the cause of apical inflammation in cases where a tooth has been, by a blow, loosened and the pulp killed, but without any decay or communication with the exterior. The difference in the odor is marked, on opening such canals, from that of canals to which microbes have access through caries. It is characteristic of carbonic acid gas, instead of the fetid sulphuretted hydrogen.

A mere mechanical stopping, like gold, does not therefore fill the requirements. Oxychloride or phosphate of zinc, while serving as good antiseptic for a time, are also poisonous to the human cell; but their antiseptic powers are soon lost, and, being somewhat porous, they absorb the secretions, and thus become a harbor for septic matter. Cotton saturated with creosote or carbolic acid is worthless, because, while irritating to the human cells, their antiseptic power is soon dissipated. Gutta-percha is a good mechanical stopping, but is not antiseptic and is difficult to thoroughly introduce. Chloro-percha, when carried into a pulp-canal and sealed up, must necessarily shrink when the chloroform evaporates, leaving the canal imperfectly filled; besides, the chloroform, vaporizing, must form a gas which theoretically would itself be an irritant.

The essential qualities for a root-filling are: 1, that it must not only be antiseptic, but must be permanently antiseptic, retaining its antiseptic powers for an indefinite length of time; 2, it should be non-escharotic, non-irritant, innocuous to the human cell; 3, impermeable to the fluids of the body; 4, non-shrinking, non-dissipating; 5, easy of introduction.

It is not so necessary that it should be a strong antiseptic as that it should be a permanent antiseptic. One of the least essential qualities is that it should be hard, for near the crown oxychloride makes a good filling to base metal fillings on.

The proper place to look for such a material is in the products of the vegetable kingdom. My reasons for thinking so are these: Animals have many ways of removing the parasites which bother them. They also have the ability to concentrate the entire fighting force of the organism at the place attacked. But trees are stationary, and the only way for them to get rid of these enemies is to secrete some substance which is poisonous to these. Probably all plants protect themselves more or less perfectly in this manner. These poisons must necessarily be such as will not injure the wood cells, and the wood cell is similar to the animal cell; so we are more likely to find a substance among these products that is at once poisonous to the microbe and innocuous to the human cell than among mineral poisons which have had no such tests. Besides, the vegetable poisons are more permanent in their character.

I have seen petrified wood where parts of the specimen impregnated with resin have preserved the wood fibres intact since the miocene epoch, while the balance has been changed to stone. These resins are principally hydrocarbons, and would naturally be good food for microbes; but with it is secreted a subtile poison that effectually keeps them away, not only from the resin itself, but from the wood cells about it. You know that amber is a fossil resin, many specimens of it show flies and insects which have been perfectly preserved since the tertiary age. A million years would be a small estimate of the time they have been there.

The reason why the coal-fields were deposited is not that vegetation was more abundant during the carboniferous age, but that the plants of that age secreted powerful and extremely permanent antiseptics, thus preserving the carbon from decay. Some of the strongest antiseptics we have now are derived from these coal products.

Antiseptics which have been distilled from vegetable compounds are not so good as the original compound, because, being separated from their original menstruum, they often lose the essential quality of permanence. The vegetable kingdom secretes almost as many different kinds of antiseptics as there are varieties of plants. Many of these secretions are not only poisonous to the microbe, but are poisonous to the human cell as well; and, therefore, should not be used. Many of these secretions, like cinchona, are good antiseptics, but soon lose their power and become worthless as a root-filling. In some the substance of the secretion dissipates, in others the antiseptic qualities deteriorate.

Some three years ago my attention was attracted to a resin which the Indians of Arizona call "balsamo del deserto." It is found in almost a fossilized state, and has withstood the ravages of time probably for centuries, perfectly preserving any substance which it may inclose. The Indians melt this and thoroughly cover any wound they may receive. I have seen the skin torn off an inch in diameter and the tissues badly bruised and lacerated covered with this material, and healing would take place by first intention, without pain, almost without swelling or the formation of a particle of pus. I have seen it applied to suppurating wounds where doubtless microbes were present, and healing take place without further pain, swelling, or discharge of pus. This shows that this material is innocuous to the human cell, and that it is an efficient germicide and antiseptic.

In filling root-canals with this material, of course nothing but long experience can fully determine its value. So far I have never known apical inflammation to recur after the root-canals were filled with it.

During the past year I have not even attempted to remove the dead pulp out of fine root-canals, simply taking that which could be extracted easily and inserting this material, and have so far had apparently better results with far less exacting labor than by the old methods. And I have every reason to believe that the treatment will be permanently successful, for I know that I am placing into these roots a material that is an efficient antiseptic, and that will *retain* its *substance* and antiseptic powers for a lifetime, and that is harmless to the human cell.

In preparing this balsam for use, I place the bottle in warm water to soften the material and then work it well into the pulp-canals. Then place a few fibres of cotton on a broach, and heat the latter over a spirit lamp about an inch back of the point. If the broach be kept slightly warmer than the tooth, the cotton will leave it and is readily worked up the canal. After the first cotton is in place I frequently saturate the additional pieces with the balsam before introducing them. The balsam is so adhesive that if this method be used for the first piece, it is liable to force a cushion of air before it. The balsam is non-irritating, and if pain be caused, it is evident too much pressure is being used, or that there is air above it. If this happens, it should be withdrawn and reinserted with more care. The canal-filling can at any time be readily removed.

The fluids of the mouth cannot permeate or deteriorate this material. It is not even necessary to fill the cavity of decay for trial canal-fillings. At the temperature of the blood the balsam is so adhesive that it will displace water and adhere to the wet surface. In chronic ulcerations, where I felt confident that tartar had collected near the apex, I have forced the material through the apical foramen and affected a cure. The theory was that the balsam would flow over the tartar and present a smooth surface, resulting in the formation of healthy tissues. I have no positive evidence that this was accomplished, as I have had no occasion to extract a tooth so treated.

In cases of abscess with fistula, I frequently cleanse the canals and fill with balsam without previous treatment, and the following phenomena are usually presented. After the lapse of a few hours there is increased apical inflammation with an additional flow of pus, accompanied, perhaps, with pain. The first thought would be that the balsam had increased the irritation, but as the parts soon heal perfectly and remain without change, I am confident the inference is fairly drawn that this increased inflammation is nature's process to effect a repair, which it is always prepared to accomplish when the parts are free from microbic life.

This is the only material that has given success in the filling of canals of temporary teeth. My former practice was to make a vent in such teeth, but now I fill the canals.

I frequently fill directly on the canal-filling with amalgam, but where gold is used the canal-filling should be covered with oxychloride.

I have had some difficulty in procuring this material on account of its locality, but should there be a demand, the supply would be abundant.

[We have, through the kindness of Dr. White, received a sample of this balsamo del deserto. Whether it will prove the long and ardently desired ideal canal-filling material remains to be proved, but all the indications point to a valuable addition to our altogether too limited list of materials.—Ed.]

